# Highly Divergent Partiti-like and Narna-like Viral RNA Sequences Detected in *Pintomyia fischeri* from São Paulo, Brazil: An Exploratory Metatranscriptomic Report

**DOI:** 10.3390/microorganisms14081723

**Published:** 2026-08-05

**Authors:** Vera Lucia Fonseca de Camargo-Neves, Antonio Charlys da Costa, Tatiana Maia de Oliveira Gonçalves, Lilian de Oliveira Guimarães, Roseane da Silva Couto, Marcos Anciete-Santos, Ramendra Pati Pandey, Vanessa Christe Helfstein, Karin Kirchgatter, Elcio Leal

**Affiliations:** 1 Instituto Pasteur, São Paulo 05403-000, SP, Brazil; vcamargo@pasteur.saude.sp.gov.br (V.L.F.d.C.-N.); lilianguimaraes@alumni.usp.br (L.d.O.G.); vchelfstein@gmail.com (V.C.H.); karink@usp.br (K.K.); 2 Instituto de Medicina Tropical, Faculdade de Medicina, Universidade de São Paulo, São Paulo 05403-000, SP, Brazil; 3 Institute of Biological Sciences, Federal University of Pará, Belém 66077-830, PA, Brazil; tatibrasilia6@gmail.com (T.M.d.O.G.); couto.roseane@gmail.com (R.d.S.C.); marcosantos@ufpa.br (M.A.-S.); elcioleal@ufpa.br (E.L.); 4 School of Biosciences and Bioengineering, D Y Patil International University (DYPIU), Akurdi, Pune 411044, Maharashtra, India; ramendra.pandey@gmail.com

**Keywords:** *Pintomyia fischeri*, *Partitiviridae*, *Narnaviridae*, metatranscriptomics, divergent viruses, sand fly

## Abstract

This study investigated the RNA virome associated with the phlebotomine sand fly *Pintomyia fischeri* using a metatranscriptomic approach applied to the Meta29 library, composed of 21 specimens collected at the São Paulo Zoo, Brazil. Read-based taxonomic analysis revealed a diverse viral community composed of viruses associated with the families *Iflaviridae*, *Narnaviridae*, *Partitiviridae*, *Reoviridae*, *Solemoviridae*, *Tombusviridae*, *Totiviridae*, and *Tymoviridae*, in addition to highly abundant unclassified RNA viruses related to the ShiM 2016 group. Assembly and annotation analyses enabled the characterization of four viral RNA genomes associated with the families *Partitiviridae* (PfPartitiV-1a-SP, PfPartitiV-1b-SP, and PfPartitiV-2-SP) and *Narnaviridae* (PfNarnaV-1-SP). Sequence comparisons revealed low amino acid identity relative to currently available reference viral sequences, supporting the classification of these sequences as highly divergent and potentially novel viral lineages. Structural modeling of the RNA-dependent RNA polymerase (RdRp) proteins identified the viral polymerase-specific catalytic motifs A, B, and C. Phylogenetic analyses further supported the evolutionary divergence of the identified viruses relative to currently described taxa. Overall, these findings expand current knowledge of viral RNA diversity associated with Neotropical sand flies and highlight the value of metatranscriptomic approaches for the detection and characterization of previously undescribed viruses associated with medically important insect vectors.

## 1. Introduction

Sand flies (Diptera: Psychodidae) play a critical role in global public health, acting as biological vectors for a wide range of pathogens, including *Leishmania* protozoa, arboviruses, and bacteria [1]. In the Neotropical region, urbanization has increasingly brought these vectors into close contact with human populations. Among them, *Pintomyia fischeri* is particularly notable in Southeastern Brazil due to its high abundance in anthropically altered environments and forest fragments within the Greater São Paulo area [2].

This species is recognized as a vector of American Cutaneous Leishmaniasis [3,4,5] and has been associated with human cases of cutaneous and mucocutaneous lesions [6,7]. More recently, its implication as a potential vector of Visceral Leishmaniasis [7,8], combined with its opportunistic feeding behavior (i.e., feeding on a wide variety of available vertebrate hosts, such as humans and domestic animals) and ecological adaptability, has reinforced the need to investigate its potential involvement in the circulation of sand fly-associated viruses [9].

Although the association between *Pi. fischeri* and Leishmaniasis has been extensively investigated, its role in the maintenance and circulation of arboviruses remains poorly understood, leaving a critical blind spot for public health and surveillance. Sand fly-borne viruses are well-known pathogens capable of causing disease in humans and non-human primates, as exemplified by the *Toscana virus* (TOSV) in the Mediterranean basin [10]. Since *Pi. fischeri* is a highly anthropophilic species that frequently colonizes peri-urban areas and forest fragments, characterizing its virome is essential to predict transmission risks. In Brazil, however, records of *Phlebovirus* and *Vesiculovirus* are largely restricted to the Amazon [11,12], leaving substantial gaps regarding viral diversity and associated transmission risks in the Central-South region.

Despite the epidemiological importance of *Pi. fischeri*, no metatranscriptomic investigation has yet characterized the virome associated with this species, and viral diversity in populations from the State of São Paulo remains unknown. This limitation is particularly relevant given the growing evidence that insect-specific viruses (ISVs) are associated with ecological interactions and may influence pathogen dynamics within vector populations. Furthermore, the detection of viruses associated with arthropods, plants, fungi, and protozoa may provide important insights into viral evolution and the ecological networks that shape host–virus interactions in complex environments, such as forest fragments, peri-urban transition zones, and anthropically modified landscapes.

To address this knowledge gap, the present study employed a metatranscriptomic approach to investigate the viral diversity associated with *Pi. fischeri* collected at the São Paulo Zoo. This site was selected as a strategic sampling area because it is a peri-urban forest fragment with high human visitation, where rich wildlife diversity creates a unique setting for vector-host interactions. While the intrinsic vector virome is classically defined by ISVs and arboviruses [13], metatranscriptomics of wild-caught individuals also captures an ‘ecological virome’—including viral sequences associated with the insects’ diet, microbiome, and environmental interactions. Thus, we hypothesized that *Pi. fischeri* harbors a highly diverse RNA virome, comprising not only intrinsic ISVs and potential arboviruses but also viruses associated with plants, fungi, and other organisms present in its habitat. In addition to identifying and characterizing viral sequences, the study quantified the relative abundance of the detected viruses, enabling the assessment of viral representativeness and distribution within the metatranscriptomic library.

By integrating qualitative and quantitative virome analyses, this work expands current knowledge of viral diversity in medically important sand flies and contributes to a broader understanding of virus–host–environment interactions. Furthermore, the identification of dominant and highly represented viral groups provides insights into the ecological structuring of viral populations associated with *Pi. fischeri*.

## 2. Materials and Methods

### 2.1. Study Sites and Sample Collection of Pi. fischeri

The sand flies were collected at the São Paulo Zoological Park (current Diretoria de Biodiversidade e Biotecnologia/DBB), located in the city of São Paulo, Brazil. The collections were carried out in 2020: 5–7 May; 6–9 October; 2–3 November; and 30 November–1 December. Specific trap locations within the zoological park were selected based on microecological criteria favorable to sand fly biology. These included proximity to animal enclosures housing potential mammalian and avian hosts, presence of preserved forest patches with dense canopy cover, and high relative humidity coupled with shaded organic soil, which serve as typical resting and breeding sites for phlebotomines. The selection of these collection points followed the methodology described previously [14,15].

Sixteen traps equipped with different light sources and carbon dioxide (CO_2_) were positioned at five dispersed points throughout the park. At each sampling point, two traps were installed at different forest strata: one at ground level (1.5 m) using white light, and another in the canopy (between 6 and 10 m high) using ultraviolet (UV) light. Both setups were baited with CO_2_. The traps remained active for 12 consecutive hours, from dusk to dawn.

**Figure 1:**
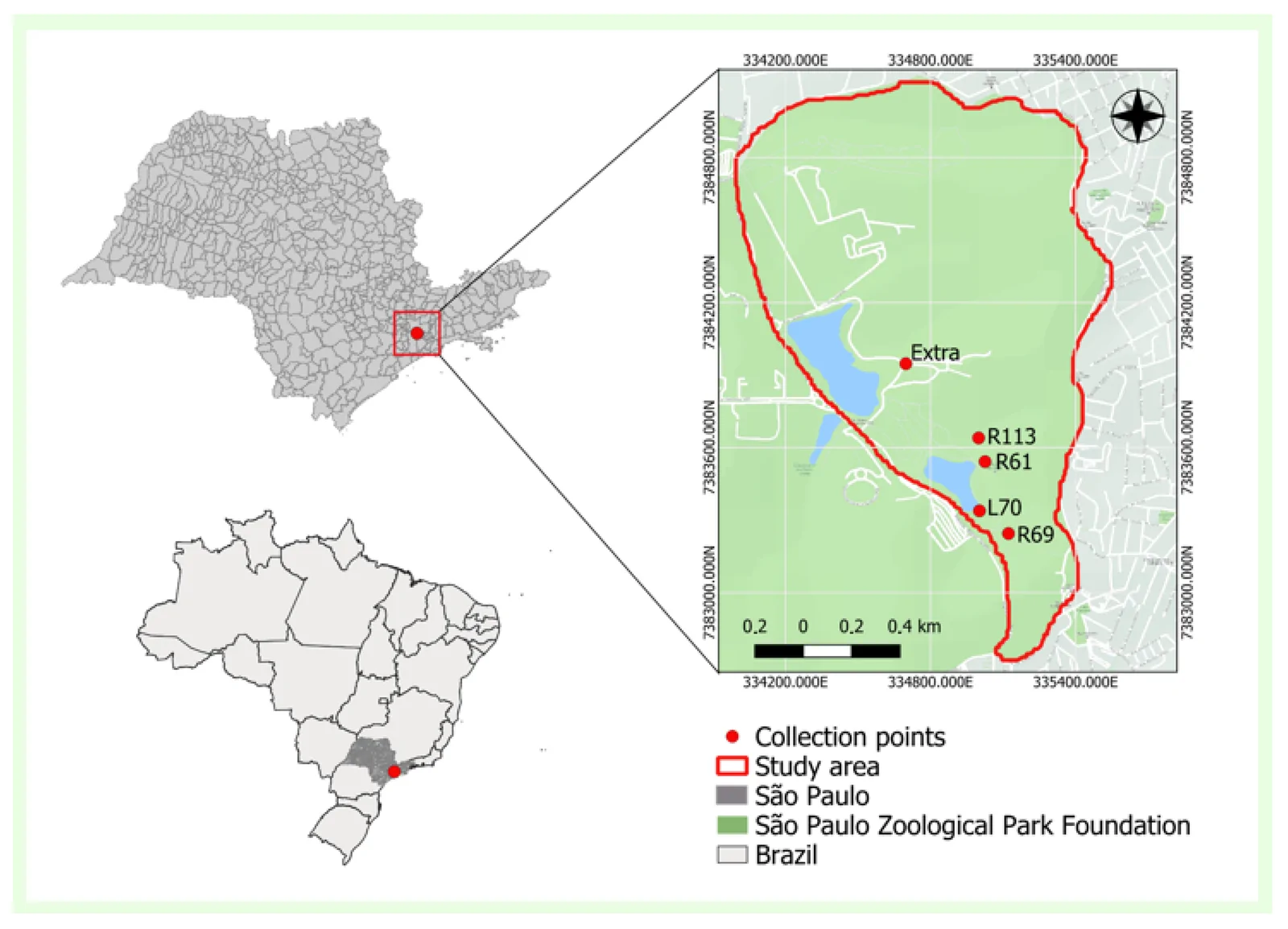
Location of collection points in the São Paulo Zoo, Brazil. Locations R61 and R69 are near a small lake and different bird enclosures; L70 is located on the lakeshore and close to the park boundary, as well as the Extra site; R113 is an area near the raptor enclosure.

The coordinates of the collection sites are site R61 (23°39′01.5″ S, 46°37′03.0″ W), located near the bird watching area; site R69 (23°39′11.2″ S, 46°36′59. 7″ W), situated near a small lake and an enclosure with several bird species; site L70 (23°39′08.1″ S, 46°37′03.9″ W), positioned on the shore of the lake, near the park boundary; site Extra (23°38′48.2″ S, 46°37′14.4″ W) outside the visitor area in a region close to buildings; and site R113 (23°38′58.3″ S, 46°37′03.9″ W) in the visitor area designated for birds of prey (Figure 1).

The captured insects were immediately placed in properly labeled cryotubes, frozen alive in liquid nitrogen, and subsequently transported to the laboratory, where they were stored in a freezer at −80 °C. Detailed information about each collection, such as the methodology used, weather conditions, dates, number of tubes collected, and duration of the collection, was documented in specific reports. Adult females that had not engorged were identified morphologically on a cold table at −20 °C, with the aid of a stereoscope, using taxonomic keys proposed by Forattini (2002) [16], Consoli and Lourenço-de-Oliveira (1994) [17], and Lane (1953) [18] at the Vector-Borne Diseases Section of the Pasteur Institute of São Paulo, Brazil.

This study was conducted in accordance with the Ethical Principles for Animal Research and approved by the Ethics Committee of the Institute of Tropical Medicine of the University of São Paulo (CPE-IMT/398A, on 8 February 2019) and the Ministry of the Environment (SISBIO No. 67527–4, on 12 April 2022).

### 2.2. RNA Extraction and Sequencing

The present study focused exclusively on the Meta29 library, which consisted of a pool of 21 *Pi. fischeri* specimens obtained from five sampling sites within the São Paulo Zoo (Figure 1), including both ground-level and canopy collections. Samples collected were pooled into a single library. This pooling strategy was adopted to ensure a sufficient mass of total RNA required for robust metatranscriptomic sequencing, focusing on providing a comprehensive characterization of the total viral diversity associated with *Pi. fischeri*. The number of individuals contributed by each sampling site is detailed in Supplementary Table S1.

The selection of the Meta29 pool was based on the results of an initial metatranscriptomic screening, in which this library exhibited the highest diversity of viral RNA signatures and contained viral contigs with low similarity to currently available reference sequences. Therefore, the objective of the present study was not to estimate viral prevalence, spatial distribution, or population-level representativeness, but rather to perform an in-depth genomic and phylogenetic characterization of novel RNA viruses associated with *Pi. fischeri*.

The pool Meta29 was homogenized in FastPrep-96 (MP Biomedicals, Santa Ana, CA, USA) for 1 min at 1500 rpm, using 900 µL of Hanks’ buffered saline solution (HBSS) and 1 g of ceramic beads. The homogenized material was centrifuged for 10 min at 14,000 rpm at 4 °C, and 500 μL of the supernatant was filtered through 0.45 µm membranes (Merck Millipore, Billerica, MA, USA). The filtrate was subjected to a mixture of nuclease enzymes containing 7 µL of TURBO DNase and 3 µL of RNase Cocktail Enzyme Mix (Thermo Fisher Scientific, Waltham, MA, USA).

RNA extraction was performed using the Maxwell 16 Viral Total Nucleic Acid Purification kit (Promega, Inc., Madison, WI, USA), according to the manufacturer’s instructions. cDNA synthesis was performed with SuperScript IV (Thermo Fisher Scientific, Waltham, MA, USA).

The second strand of cDNA was synthesized using DNA Polymerase I Large (Klenow) Fragment (Promega Inc., Madison, WI, USA). The DNA library was prepared using the Nextera XT DNA Library Preparation Kit (Illumina Inc., San Diego, CA, USA), following the manufacturer’s recommendations. Sequencing was performed on the Illumina MiSeq platform using paired-end sequencing chemistry (2 × 150 bp) at the Central Laboratory of the Hospital das Clínicas of the University of São Paulo.

### 2.3. Bioinformatics Analysis

Raw reads were processed with Trimmomatic v0.39 [19] for adapter removal and quality trimming. Adapter sequences were removed using the ILLUMINACLIP module (NexteraPE-PE.fa:2:30:10:8:true), and low-quality bases were trimmed with the SLIDINGWINDOW:4:30 parameter. Reads shorter than 50 bp after trimming were discarded. Sequencing quality before and after trimming was assessed with FastQC v0.12.1 [20], including read length distribution, GC content, and per-base sequence quality. Raw sequencing data comprised 633,564 reads (170.9 Mb), whereas 529,596 reads (64.4 Mb) were retained after trimming and quality filtering, exhibiting Phred quality scores predominantly above Q30. Host depletion was not performed because no complete reference genome is currently available for *Pi. fischeri*.

De novo virome assemblies were generated using rnaviralSPAdes v3.15.5 [21], optimized for RNA virus reconstruction from transcriptomic datasets, using default parameters in Galaxy Europe Server (https://usegalaxy.eu/login/start; accessed on 10 August 2025). Redundant contigs were subsequently clustered using CD-HIT-EST v4.8.1 [22] at ≥98% nucleotide identity, retaining representative sequences for downstream analyses.

To evaluate relative viral abundance and coverage, quality-filtered reads from the Meta29 library were mapped against a custom viral reference composed of the viral genomic sequences assembled in this study (PfNarnaV-1-SP, PfPartitiV-1a-SP, PfPartitiV-1b-SP, and PfPartitiV-2-SP). Read alignment was performed using Bowtie2 v2.5.5 [23] (Galaxy Version 2.5.5 + galaxy0) in end-to-end mode with the very-sensitive preset. SAM/BAM files were processed, sorted, and indexed using Samtools v1.11 [24]. Coverage statistics, mapping quality metrics, and duplication rates were obtained using Qualimap 2 BamQC [25] (--collect-overlap-pairs, -nw 400, --paint-chromosome-limits, -hm 3, and --skip-duplicated). Per-position coverage profiles were generated using SAMtools depth [24] to assess coverage uniformity across the full length of each viral genome. Read quantification was performed using featureCounts (Subread package; Galaxy Version 2.1.1 + galaxy0) [26]. Gene models were generated in GTF format from coding sequence (CDS) annotations predicted with Prokka v1.14.6 using the “Viruses” mode (--kingdom Viruses, --gcode 1, e-value 1 × 10^−6^) [27]. Read counting was restricted to CDS regions (-t CDS) using the “ID” attribute (-g ID, --minOverlap 1, -Q 0, -s 1). Viral abundance was normalized using the RPKM (Reads Per Kilobase of transcript per Million mapped reads) metric, calculated based on CDS length and the 529,596 retained reads, to estimate the relative representation of each viral genome within the library [28].

**Figure 2:**
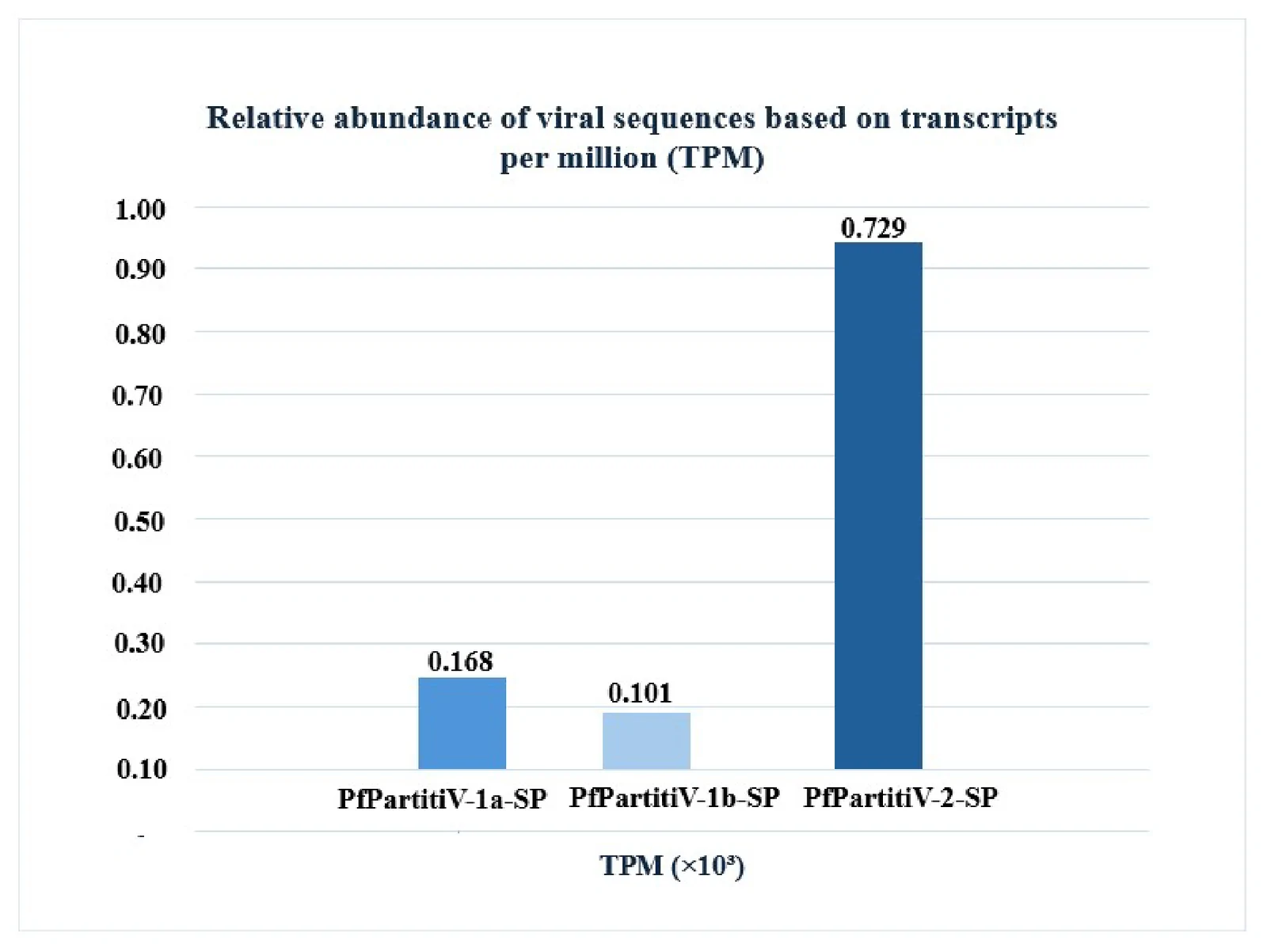
Relative abundance of the assembled viral genomes detected in the Meta29 metatranscriptomic library of *Pintomyia fischeri*. Viral abundance was estimated by remapping sequencing reads to each assembled viral genome and normalized as transcripts per million (TPM), accounting for differences in genome length and sequencing depth. Only assembled viral genomes supported by read remapping were included in the abundance analysis. TPM values represent relative transcript abundance rather than absolute viral genome abundance because the sequencing library was generated using a total RNA metatranscriptomic approach.

To identify and characterize the RNA virome at both the read and contig levels, all assembled sequences and quality-filtered reads were screened using DIAMOND v2.1.9 [29] in BLASTx mode against a curated RNA-dependent RNA polymerase (RdRp) database from the Serratus project (https://serratus.io; accessed on September 2025). Searches were performed using default sensitivity settings, the BLOSUM62 substitution matrix, tantan masking, bidirectional strand search, and an e-value threshold of 1 × 10^−3^, reporting up to 25 matches per query. No minimum identity or alignment coverage thresholds were applied during this initial screening to maximize sensitivity for highly divergent RNA viruses. Read-level taxonomic profiles were visualized using MEGAN Community Edition v6.25.10 for hierarchical classification (Figure 2) [30]. Contig-level taxonomic assignments were determined based on the best-supported hit, considering bit-score, alignment coverage, and taxonomic consistency.

To refine viral annotation, contigs were additionally compared against the NCBI RefSeq Viral protein database using BLASTx and BLASTp (https://blast.ncbi.nlm.nih.gov/Blast.cgi; accessed on 12 August 2025) [31]. Reference sequences from the *Narnaviridae* and *Partitiviridae* families were included following ICTV recommendations (accessed on 13 August 2025) [32] to support taxonomic confirmation. Viral genomic segments were aligned using MAFFT v7 [33], and the RdRp ORF was predicted using ORFfinder v0.4.3 (accessed on 12 August 2025) [34], configured with the standard genetic code, a minimum ORF length of 75 nucleotides, and restricting the search to open reading frames initiated strictly with the ATG start codon. Conserved RdRp domains and motifs were identified using the Conserved Domains Tool v0.4.4 [35] and Motif Finder (https://www.genome.jp/tools/motif/, accessed on 12 August 2025) [36]. Novel virus candidates were validated using LucaProt [37] and PalmAnnot (https://github.com/rcedgar/palm_annot; accessed on 20 September 2025) [38].

### 2.4. Homology Modeling of RdRp Proteins

The amino acid sequences of the RNA-dependent RNA polymerases (RdRps) from PfPartitiV-1a-SP [PX992747], PfPartitiV-1b-SP [PX992748], PfPartitiV-2-SP [PX992749], and PfNarnaV-1-SP [PX992750], available in GenBank, were individually submitted to the SWISS-MODEL platform (https://www.swissmodel.expasy.org/interactive, accessed on 20 September 2025) [39] for three-dimensional structural prediction by homology modeling.

For each RdRp, sequence–template alignments were generated using the HHblits algorithm, and structural models were built with ProMod3 v3.6.0. Template selection was automatically performed by the SWISS-MODEL (https://swissmodel.expasy.org/interactive; accessed on 12 June 2026) [39] server based on sequence similarity, structural coverage, and template quality, using experimentally resolved structures available in the Protein Data Bank (PDB). Model quality was assessed using the default SWISS-MODEL scoring metrics, including GMQE and QMEANDisCo. Molecular visualization and mapping of the conserved catalytic motifs A, B, and C were performed using UCSF Chimera v1.17 [40].

### 2.5. Phylogenetic Analysis

Phylogenetic analyses were performed exclusively using amino acid sequences of the RNA-dependent RNA polymerase (RdRp). Multiple sequence alignments were generated with MAFFT v7.520 using the L-INS-i algorithm [33]. Maximum-likelihood phylogenetic trees were inferred with IQ-TREE v2.2.6 under the best-fit substitution model selected by ModelFinder (https://iqtree.github.io/; accessed on 28 July 2026) [41], with branch support assessed using 1000 ultrafast bootstrap replicates [42].

The resulting phylogenetic trees were visualized and edited in FigTree v1.4.2 (http://tree.bio.ed.ac.uk/software/figtree/; accessed on 28 September 2025) [43]. The sequencing data generated from the Meta29 sample are publicly deposited in the NCBI Sequence Read Archive (SRA) under BioProject PRJNA1465927, BioSample SAMN59824430, and SRA SRR38603290. The sequences of the RdRp coding regions of the four viruses identified in this study are available in GenBank under the accession numbers PfPartitiV-1a-SP (PX992747), PfPartitiV-1b-SP (PX992748), PfPartitiV-2-SP (PX992749), and PfNarnaV-1-SP (PX992750).

## 3. Results

### 3.1. Viral Mapping and Abundance Analysis

Sequencing of the Meta29 library generated 633,564 raw reads, totaling 170.9 Mb of data. After adapter removal and quality filtering, 529,596 reads (83.6% retention), corresponding to 64.4 Mb, were retained for downstream analyses.

Remapping of the filtered reads against the four assembled viral genomes resulted in 26,909 aligned reads, corresponding to 5.08% of the processed dataset (Supplementary Table S2). Alignment quality metrics indicated a mean mapping quality of 30.09, a global mean coverage depth of 964.10×, and an estimated error rate of 2.99%. The mean GC content was 43.55%, while the duplication rate reached 77.55%, suggesting low library complexity and the overrepresentation of a subset of highly abundant viral sequences.

Abundance estimates obtained through taxonomic classification and genome remapping are not directly comparable. Taxonomic classification was performed using individual reads queried against reference databases, whereas remapping statistics consider only reads aligned to the four viral genomes recovered after de novo assembly. Thus, reads assigned to certain viral groups may correspond to fragmented sequences, low-abundance viruses that were not assembled, or genomic regions not represented in the final contigs. Consequently, differences between counts obtained by both approaches are expected and do not represent numerical inconsistencies.

Coverage analyses revealed marked differences in the relative abundance of the recovered viruses. Among the four assembled genomes, PfPartitiV-2-SP showed the highest representation, with 5,095,434 mapped bases, a mean coverage of 3095.65×, and 9875 reads assigned by featureCounts. The genomes PfPartitiV-1a-SP and PfPartitiV-1b-SP exhibited mean coverages of 734.89× and 452.49×, with 2358 and 1391 assigned reads, respectively.

RPKM values corroborated this abundance pattern, confirming PfPartitiV-2-SP as the most abundant virus in the library (12,582.98), followed by PfPartitiV-1a-SP (2998.29), PfPartitiV-1b-SP (1769.26), and PfNarnaV-1-SP (27.37) (Supplementary Table S2).

In the case of PfNarnaV-1-SP, although only 31 reads were assigned to the viral CDS by featureCounts, remapping against the complete genome resulted in 21,210 aligned bases and a mean coverage of 8.95× across 2369 nt. This difference arises because featureCounts quantifies only reads overlapping annotated coding regions, whereas coverage statistics include all bases aligned to the viral genome. This value represents the sum of coverage depth across the genome, not the length of individual reads.

Aligned reads were distributed across nearly the entire genomic sequence, covering 2364 of 2369 positions (99.8%), with a mean depth of 8.97×, supporting the recovery of a near-complete genome. Although the coverage depth was lower than that observed for the other viruses, the extensive positional coverage provides additional support for the recovery and characterization of PfNarnaV-1-SP.

Overall, the results revealed marked differences in the relative abundance of the recovered viruses, with a predominance of sequences belonging to the *Partitiviridae* family, particularly PfPartitiV-2-SP, while PfNarnaV-1-SP showed lower representation. These differences may reflect both the biological dynamics of viruses associated with *Pi. fischeri* and limitations inherent to the sequencing depth employed.

### 3.2. Viral Abundance Based on Normalized Transcript Counts

The abundance of the assembled viral genomes recovered from the Meta29 metatranscriptomic library was estimated by remapping sequencing reads to each assembled genome and calculating transcript abundance as transcripts per million (TPM), thereby accounting for differences in genome length and sequencing depth.

Three partitiviruses dominated the assembled virome. PfPartitiV-2-SP was the most abundant viral genome, accounting for 729,052.63 TPM (72.92% of the total normalized viral transcripts), followed by PfPartitiV-1a-SP (168,259.58 TPM; 16.80%) and PfPartitiV-1b-SP (101,097.87 TPM; 10.12%). In contrast, PfNarnaV-1-SP exhibited substantially lower transcript abundance (1589.92 TPM; 0.16%), despite being recovered as a complete viral genome through de novo assembly and supported by read remapping (Figure 2; Table 1).

Because the sequencing library was generated using a total RNA metatranscriptomic approach, TPM values represent the relative abundance of viral transcripts rather than absolute viral genome copy numbers. Consequently, these estimates may be influenced by viral transcriptional activity, genome organization, RNA stability, and biases associated with library preparation and sequencing.

Only assembled viral genomes supported by read remapping were included in the abundance analysis presented in Figure 2 and Table 1. In contrast, low-confidence taxonomic assignments derived solely from individual read similarity searches were excluded to avoid overinterpretation of extremely low-abundance signals.

Assigned reads correspond to sequencing reads remapped to the assembled viral genomes and quantified using featureCounts. Genome length is reported in base pairs (bp) and kilobases (kb; bp/1000). RPK (Reads Per Kilobase) was calculated by normalizing read counts according to genome length, whereas TPM (Transcripts Per Million) further accounts for sequencing depth, allowing comparisons of relative viral transcript abundance among assembled viral genomes. Detailed mapping statistics and genome coverage metrics are provided in Supplementary Tables S2 and S3.

### 3.3. Novel Virus Discovery

#### 3.3.1. Genome Characterization

A total of four viral sequences (PfPartitiV-1a-SP, PfPartitiV-1b-SP, PfPartitiV-2-SP, and PfNarnaV-1-SP) were identified in the sand fly *Pi. fischeri*. Similarity searches were performed at both nucleotide and amino acid levels using BLASTn and BLASTp against sequences available in the NCBI databases (Supplementary Table S4). All viral sequences corresponded to complete or near-complete genomic segments containing the RdRp-coding region, enabling downstream comparative and phylogenetic analyses.

The PfNarnaV-1-SP genome comprises 2369 nucleotides (nt) and contains two open reading frames (ORFs). ORF1 encodes an RNA-dependent RNA polymerase (RdRp) of 786 amino acids (aa), in which the Mitovirus RNA-dependent RNA polymerase domain (Mitovir_RNA_pol) was identified, including the conserved catalytic motifs A and B characteristic of the family *Narnaviridae*.

ORF2 encodes a putative viral protein (VP1) of 712 aa associated with the DUF7103 domain, which is currently classified as a domain of unknown function. BLASTp analysis revealed similarity to *Orius laevigatus narnavirus* 1 (GenBank: XGU09087.1; *Orius laevigatus*, Spain) [44], with 92% query coverage and 44.59% amino acid identity (Supplementary Table S4). The combination of high alignment coverage and relatively low amino acid identity suggests that PfNarnaV-1-SP represents an evolutionarily divergent lineage within the family *Narnaviridae*.

The PfPartitiV-1a-SP (1703 nt) and PfPartitiV-1b-SP (1672 nt) sequences exhibited highly similar genomic organizations, differing mainly in genome length. Both genomes contain a single ORF encoding a 494 aa RdRp associated with the conserved RdRP_1 and RdRP_4 domains, as well as the canonical catalytic motifs A, B, and C characteristic of the family *Partitiviridae* (Supplementary Table S4).

BLASTp searches identified sequence WWV86526.1 (GenBank; detected in *Rhinolophus pearsonii*, China, collected between 2015–2017) as the closest match, sharing amino acid identities of 53.23% and 52.80% and query coverages of 87% and 89%, respectively. The relatively low amino acid identities despite the extensive alignment coverage indicate substantial evolutionary divergence from currently available reference sequences, suggesting that these viruses represent distinct and highly divergent partiti-like lineages (Supplementary Table S4).

The PfPartitiV-2-SP genome, comprising 1646 nt, also contains a single ORF encoding a 493 aa RdRp associated with the RdRP_1 and RdRP_4 domains. Similarity analyses identified the closest match as *Hubei partiti-like virus* 57 (APG78229.1), originally detected in *Odonata* from China in 2013 [45], with 89% query coverage and 53.25% amino acid identity. As observed for PfPartitiV-1a-SP and PfPartitiV-1b-SP, the combination of extensive alignment coverage and relatively low amino acid identity supports the classification of PfPartitiV-2-SP as a highly divergent partiti-like virus.

Collectively, these findings expand current knowledge of RNA viruses associated with *Pi. fischeri* and reveal the presence of evolutionarily divergent viral lineages related to the families *Partitiviridae* and *Narnaviridae*. The low amino acid identities observed in comparison with publicly available sequences, together with their distinct phylogenetic placement, support the hypothesis that these viruses represent previously undescribed viral taxa circulating in Neotropical phlebotomine sand flies, an insect group that remains largely unexplored from a viromic perspective.

#### 3.3.2. Structural Modeling of RdRp Proteins

Homology modeling of the RdRp proteins from PfPartitiV-1a-SP, PfPartitiV-1b-SP, PfPartitiV-2-SP, and PfNarnaV-1-SP generated structural models with architectures consistent with RNA-dependent RNA polymerases, despite the low sequence identity relative to available templates.

The models generated for PfPartitiV-1a-SP, PfPartitiV-1b-SP, and PfPartitiV-2-SP showed approximately 82% structural coverage, with GMQE values ranging from 0.40 to 0.42 and QMEANDisCo Global scores between 0.47 ± 0.05 and 0.50 ± 0.05, indicating moderate structural confidence. Sequence identity relative to the selected templates ranged from 13.15% to 14.64%.

In contrast, the PfNarnaV-1-SP model exhibited lower structural coverage (24%), with a GMQE value of 0.12 and a QMEANDisCo Global score of 0.46 ± 0.06, indicating limited structural reliability. Therefore, structural interpretations derived from this model should be considered preliminary. Nevertheless, the conserved RdRp motifs A, B, and C were independently identified through PalmAnnot and sequence-based analyses and were subsequently mapped onto the predicted structures for visualization purposes [38].

Overall, the taxonomic assignment of the identified viruses was corroborated by multiple independent lines of evidence, including sequence similarity searches, detection of conserved RdRp motifs, validation using LucaProt, PalmAnnot and phylogenetic inference. Structural modeling was employed as a complementary tool for visualization of the predicted RdRp architecture, particularly for the *Partitiviridae*-associated viruses, and not as a basis for functional inference [37,38].

#### 3.3.3. Description of the RdRp Domain

The PfPartitiV-1a-SP, PfPartitiV-1b-SP, PfPartitiV-2-SP, and PfNarnaV-1-SP sequences encode RNA-dependent RNA polymerase (RdRp) proteins, enzymes essential for the replication of positive-sense single-stranded RNA viruses (+ssRNA) [46].PfPartitiV-1a-SP, PfPartitiV-1b-SP, and PfPartitiV-2-SP contain two domains: viral RNA-dependent RNA polymerase (RdRP_1, PF00680), and viral RNA-directed RNA polymerase (RdRP_4, PF02123). RdRP_1 corresponds to the catalytic region responsible for the synthesis of new RNA strands from the genomic template, while RdRP_4 acts in maintaining the enzymatic conformation and controlling replicative fidelity (Supplementary Table S5).

The RdRP_4 domain, conserved in RNA viruses that do not have a DNA intermediate in their cycle, is essential for the synthesis of complementary RNA [46,47]. The replication occurs through a biphasic mechanism [48]. In the first phase, called initiation, RNA synthesis is initiated at the 3′ end of the template or in adjacent regions through a primer-independent (de novo) process. This process involves the binding of a nucleotide triphosphate (NTP) to the 3′-OH end of the initiator NTP. In the subsequent phase, called elongation, NTPs are sequentially added, forming the complementary RNA strand through repeated nucleotide transfer reactions [48].

The PfNarnaV-1-SP sequence exhibited a distinct genomic organization characterized by the presence of the Mitovirus RNA-dependent RNA polymerase domain (Mitovir_RNA_pol, PF05919) and the DUF7103 domain (PF23396), currently annotated as a domain of unknown function. The Mitovir_RNA_pol domain has been previously reported in members of the family *Narnaviridae*, particularly in viruses related to the genus *Mitovirus* [49]. In contrast, the DUF7103 domain remains poorly characterized, and no experimentally validated biological function has yet been established for proteins containing this domain.

#### 3.3.4. Description of the Domain’s Motifs

**Figure 3:**
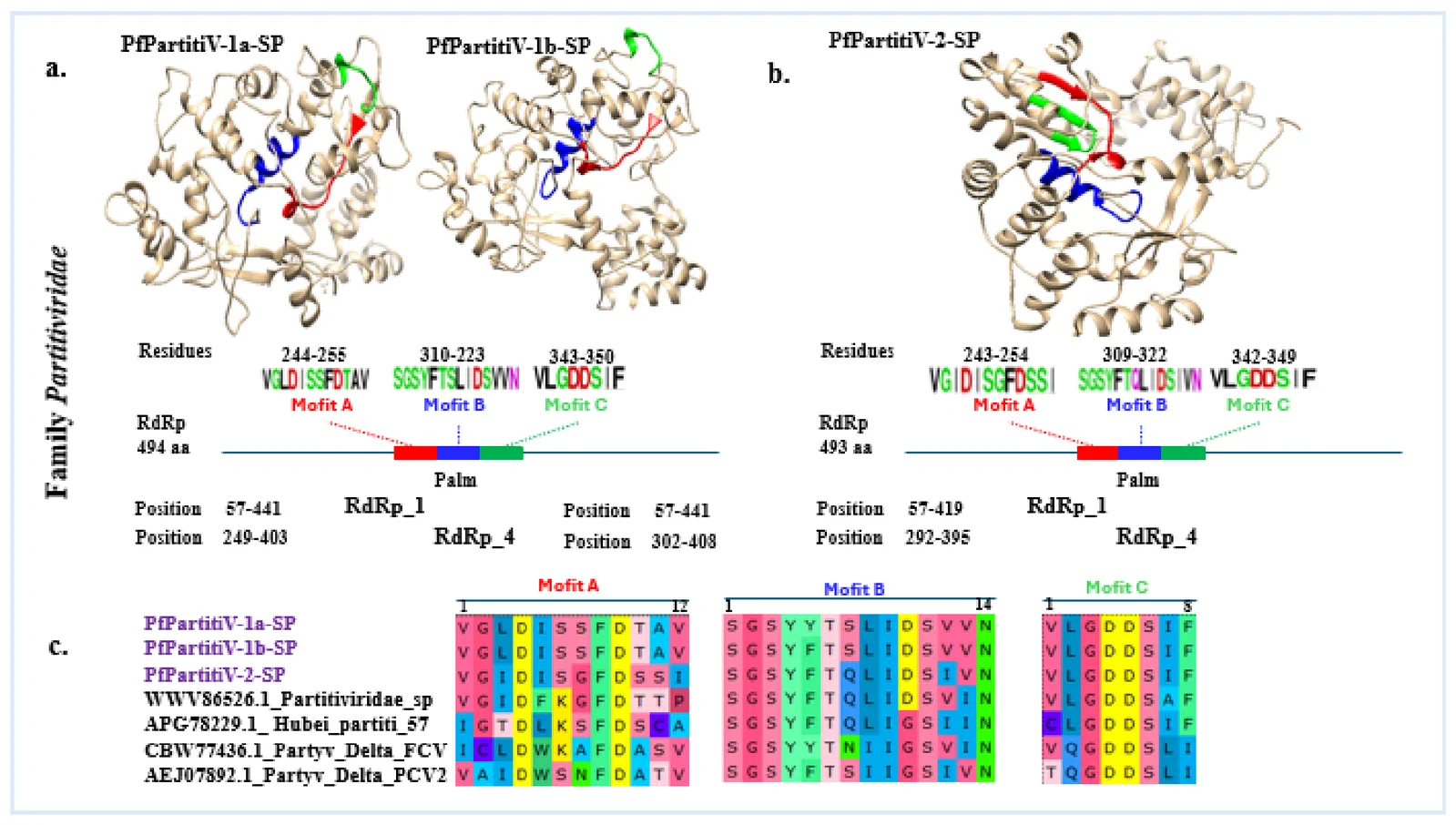
Structural modeling and comparative analysis of the catalytic motifs of RNA-dependent RNA polymerases (RdRps) from PfPartitiV viruses associated with the sand fly *Pi. fischeri* (*Partitiviridae*). (**a**). Three-dimensional structures of RdRps PfPartitiV-1a-SP and PfPartitiV-1b-SP, with catalytic motifs highlighted: Motif A (residues 244–255, red), Motif B (residues 310–323, blue), and Motif C (residues 343–350, green), located within the catalytic palm domain between RdRP_1 and RdRP_4. (**b**). Three-dimensional structure of RdRp PfPartitiV-2-SP showing Motif A (243–254), Motif B (309–322), and Motif C (342–349), also positioned in the palm domain. (**c**). Multiple sequence alignment of PfPartitiV-1a-SP, PfPartitiV-1b-SP, and PfPartitiV-2-SP showing the initial consensus sequences: Motif A (VGxDISxFDxxx), B (SGSYxTxLIDSxVN), and C (VLGDDSIF). Comparison with representative *Partitiviridae* members (WWW86562.1, AGP78292.1, CBW774361.1, AEJ07892.1) revealed broader conserved patterns: Motif A (xxxDxxxFDxxx), Motif B (SGSYxTxxIxSxxN), and Motif C (xxGDDSxx). Motifs A, B, and C are highlighted in red, blue, and green, respectively, and the WebLogo representation illustrates residue frequency and conservation at each position, emphasizing central catalytic residues.

The structural and comparative characterization of the PfPartitiV-1a-SP, PfPartitiV-1b-SP, PfPartitiV-2-SP, and PfNarnaV -1-SP RNA-dependent RNA polymerase (RdRp) sequences of viruses associated with the sand fly *Pi. fischeri* showed significant conservation of catalytic motifs A, B, and C among representatives of the *Partitiviridae* and *Narnaviridae* families. Three-dimensional models of the proteins show that these motifs are organized in the catalytic palm domain, with similar positioning among viral variants (Figure 3a,b).

In the comparative analysis, it was observed that the RdRps of the PfPartitiV-1a-SP, PfPartitiV-1b-SP, and PfPartitiV-2-SP viruses have the RdRP_1 and RdRP_4 domains, in addition to the essential catalytic motifs: Motif A (VGLDISSFDTAV and VGIDISGFDSSI, residues 244–255 and 243–254, respectively), Motif B (SGSYYTSLIDSVVN and SGSYFTQLIDSIVN, residues 310–323 and 309–322), and Motif C (VLGDDSIF and VLGDDSIF, residues 343–350 and 342–349).

This motif, together with consensuses from different members of the *Partitiviridae* family, such as WWW86562.1_Partitiviridae_sp, AGP78292.1_Hubei_partiti_57, CBW774361.1_Party_Delta_FCV, and AEJ07892.1_Party_Delta_PCV2, resulted in the following conserved patterns: A (xxxDxxxFDxxx), B (SGSYxTxxIxSxxN), and C (xxGDDSxx) (Figure 3c).

Thus, the graphical representation of WebLogo (Figure 3c) highlights the frequency and conservation of amino acid residues at specific positions throughout multiple aligned sequences, reinforcing the functional importance of these regions in the enzymatic activity of RdRps.

**Figure 4:**
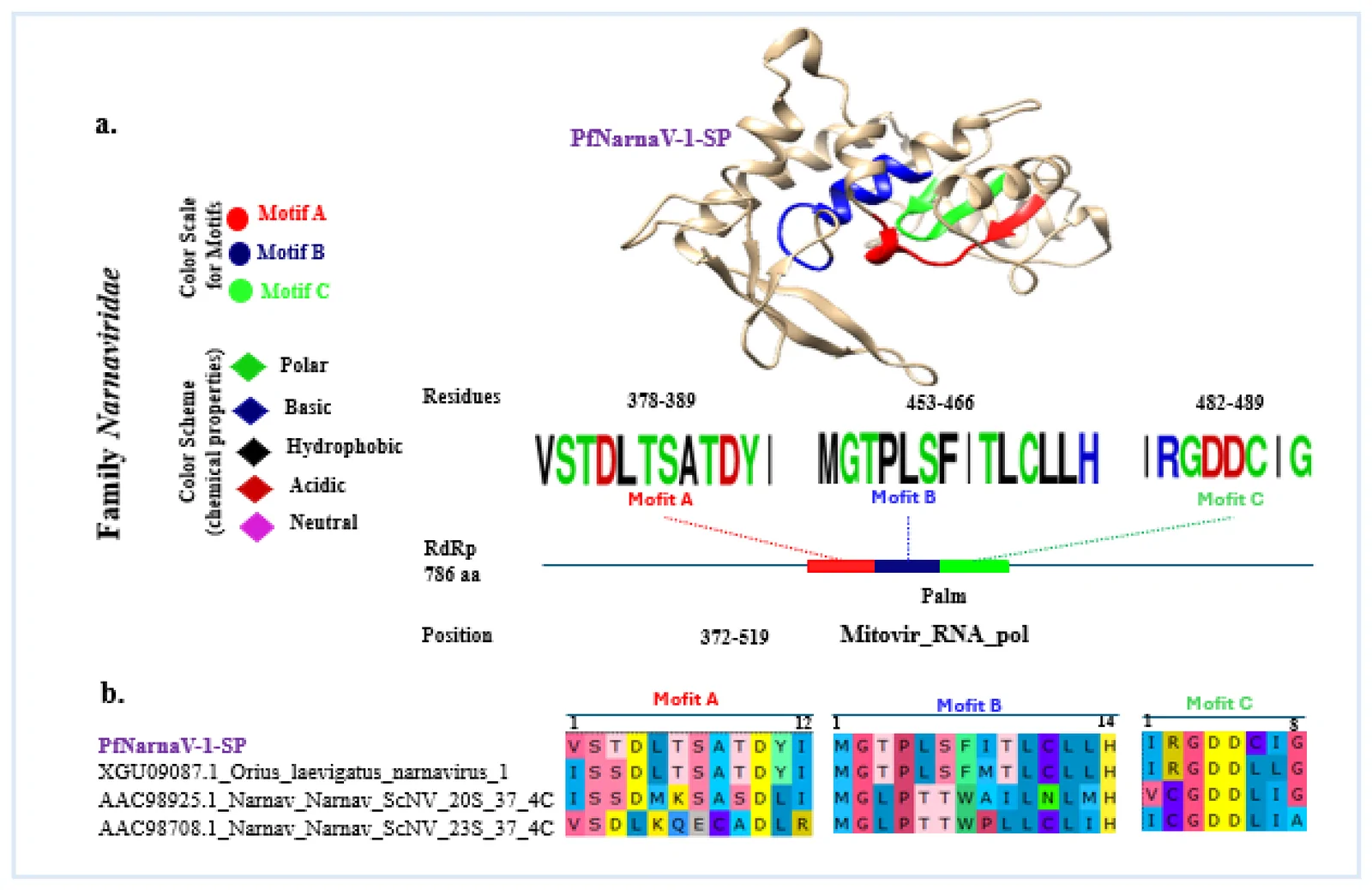
Structural modeling and comparative analysis of the catalytic motifs of PfNarnaV-1-SP RdRp (*Narnaviridae*). (**a**). Three-dimensional structure of the RdRp highlighting motifs A (VSTDLTSATDYI, red, residues 378–389), B (MGTPLSFITLCLLH, blue, residues 453–466), and C (IRGDDCIG, green, residues 482–489), located in the palm domain. (**b**). Multiple sequence alignment with *Narnaviridae* representatives showing conserved motifs visualized using WebLogo: Motif A (xSxxxxxxxDxx), Motif B (MGxPxxxxxLxLxH), and Motif C (xxGDDxxxx). Each logo displays stacks of symbols representing sequence conservation and the relative frequency of amino acids at each position, providing a detailed representation of motifs A, B, and C beyond simple consensus sequences.

In contrast to viruses of the *Partitiviridae* family, the RdRp protein of the PfNarnaV-1-SP virus, belonging to the *Narnaviridae* family, presents a distinct three-dimensional structure, evidenced by ribbon modeling, in which the catalytic motifs A, B, and C are distributed in specific regions and highlighted by circles (Figure 4). This structure is organized around the Mitovir_RNA_pol domain, which spans residues 372–619 and corresponds to the functional region of RNA polymerase. The identified conserved motifs include Motif A (VSTDLTSATDYI, residues 378–389), Motif B (MGTPLSFITLCLLH, residues 453–466), and Motif C (IRGDDCIG, residues 482–489) (Figure 4a). Comparison with RdRps from other representatives of the same family, for example, XGU09087.1_Orius laevigatus narnavirus 1, AAC98925.1_Narnavirus ScNV 20S 37 4C, and AAC98708.1_Narnavirus ScNV 23S 37 4C. These conserved patterns are Motif A (xSxxxxxxxDxx), Motif B (MGxPxxxxLxLxH), and Motif C (xxGDDxxxx) (Figure 4b).

These consensuses, graphically represented through WebLogo and multiple alignment, highlight the functional conservation of catalytic residues in a specific position, reinforcing the structural and enzymatic importance of RdRps from the *Narnaviridae* family. To understand the functional particularities of these regions, each catalytic motif was examined individually, highlighting its structural relevance and the degree of conservation among viral families.

Motif A contains conserved aspartate residues that are known to coordinate divalent metal ions (Mg^2+^ or Mn^2+^) during RNA synthesis, playing a central role in the catalytic activity of RNA-dependent RNA polymerases [50,51,52]. The sequences identified in PfPartitiV-2-SP (VGIDISGFDSSI) and PfPartitiV-1a/1b-SP (VGLDISSFDTAV) retain the characteristic aspartate-containing core observed in other members of the family. Similarly, PfNarnaV-1-SP (VSTDLTSATDYI) preserves the conserved catalytic aspartate residue despite sequence divergence, indicating conservation of this hallmark RdRp feature.

Motif B is commonly associated with nucleotide triphosphate (NTP) recognition and positioning within the polymerase active site. Conserved aromatic and polar residues, including tyrosine, phenylalanine, and threonine, contribute to substrate interaction and polymerase [53,54]. The motifs identified in PfPartitiV-2-SP (SGSYFTQLIDSIVN), PfPartitiV-1a-SP (SGSYYTSLIDSVVN), and PfPartitiV-1b-SP (SGSYFTSLIDSVVN) exhibited high similarity to those reported for other partitiviruses. In contrast, PfNarnaV-1-SP displayed a distinct motif composition, consistent with the evolutionary divergence between members of the families *Partitiviridae* and *Narnaviridae*.

Motif C contains the highly conserved GDD catalytic core that is characteristic of RNA-dependent RNA polymerases and is directly involved in phosphodiester bond formation during RNA elongation [52,55,56]. The motif VLGDDSIF identified in PfPartitiV-1a-SP, PfPartitiV-1b-SP, and PfPartitiV-2-SP, as well as the motif IRGDDCIG identified in PfNarnaV-1-SP, retain the canonical GDD signature. The conservation of these catalytic residues provides additional evidence supporting the annotation of these proteins as viral RNA-dependent RNA polymerases.

### 3.4. Phylogenetic Trees 

In total, four viral RdRp protein sequences were identified in metatranscriptomic libraries generated from *Pi. fischeri* specimens collected at the São Paulo Zoo (DBB). For comparative and taxonomic purposes, these sequences were analyzed individually against reference RdRp sequences, including 17 sequences from the family *Narnaviridae* and 36 from *Partitiviridae*, as well as related unclassified viral sequences available in public databases.

Maximum likelihood phylogenetic inference reconstructed well-supported taxonomic groupings consistent with current classifications within both families. According to the Bayesian Information Criterion (BIC) [57], ModelFinder selected LG+F+R4 and VT+F+I+G4 as the best-fitting amino acid substitution models for *Narnaviridae* and *Partitiviridae*, respectively.

**Figure 5:**
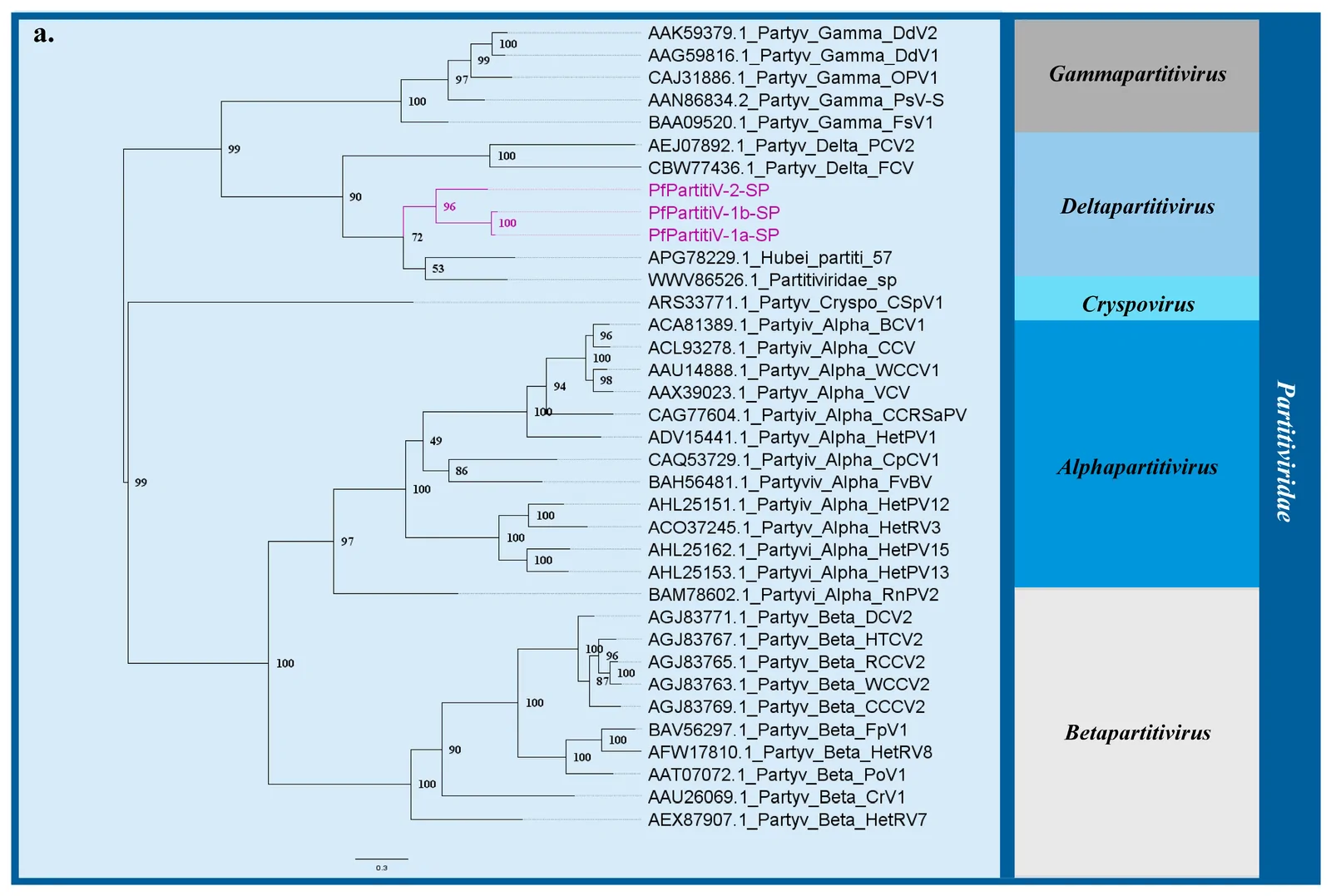

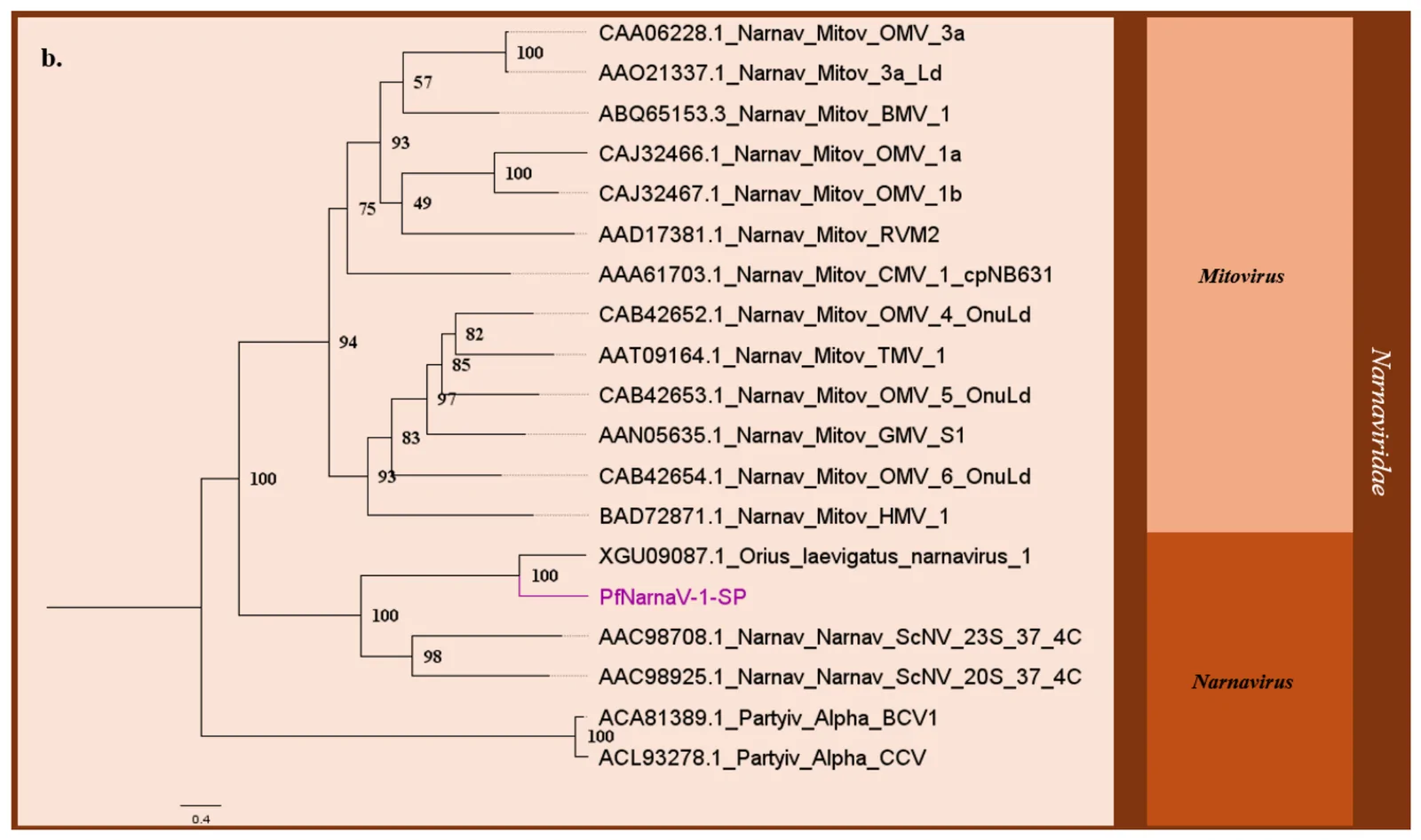
Maximum likelihood phylogenetic trees inferred from RNA-dependent RNA polymerase (RdRp) amino acid sequences of viruses belonging to the families *Partitiviridae* and *Narnaviridae*. Phylogenetic analyses were performed using IQ-TREE, with the best-fit substitution models selected by ModelFinder: VT+F+I+G4 for *Partitiviridae* (**a**) and LG+F+R4 for *Narnaviridae* (**b**). Branch support was estimated using 1000 ultrafast bootstrap replicates, and only bootstrap values ≥70% are shown at the nodes. Viral sequences identified in this study are highlighted in purple (PfPartitiV-1a-SP, PfPartitiV-1b-SP, PfPartitiV-2-SP, and PfNarnaV-1-SP).

Three of the identified sequences clustered robustly within the family *Partitiviridae* (Figure 5a) and were designated as: *Pi. fischeri partitivirus* 1-SP (PfPartitiV-1a-SP and PfPartitiV-1b-SP) and *Pi. fischeri partitivirus* 2-SP (PfPartitiV-2-SP). The remaining sequence grouped within the family *Narnaviridae* (Figure 5b) and was designated as *Pi. fischeri narnavirus* 1-SP (PfNarnaV-1-SP).

BLASTp searches further supported these phylogenetic placements. Sequences PfPartitiV-1a-SP, PfPartitiV-1b-SP, and PfPartitiV-2-SP shared their highest amino acid similarities with previously described partiti-like viruses, such as *Hubei partiti-like virus 57* and sequence WWV86526.1, exhibiting identity values of 53.27%, 52.83%, and 53.25%, respectively. Conversely, PfNarnaV-1-SP showed 93% query coverage and 44.59% amino acid identity to *Orius laevigatus narnavirus* 1.

The taxonomic identity of these novel lineages was independently confirmed by the machine-learning and catalytic-signature tools LucaProt [37] and PalmAnnot [38]. Furthermore, structural analysis revealed the presence of characteristic and functionally conserved domains and catalytic motifs of RdRp in all sequences. Taken together, the convergence of phylogenetics, local similarity profiles, protein domain integrity, and independent machine-learning classifications consistently supports the assignment of these sequences as divergent viral lineages within *Partitiviridae* and *Narnaviridae*.

## 4. Discussion

In this study, we used a metatranscriptomic approach to characterize the virome of *Pi. fischeri* collected at the São Paulo Zoo. The viral community identified revealed a predominance of RNA viruses associated not only with arthropods but also with plants, fungi, and protists. This diversity likely reflects the species’ feeding habits and life cycle. When compared to existing literature on sand fly viromes, our findings align with a growing body of evidence showing that phlebotomine sand flies harbor highly diverse communities. Metatranscriptomic studies in both the Old World (e.g., *Phlebotomus* species in Europe and Asia) and the Neotropics (e.g., *Lutzomyia longipalpis* and other species in Brazil) have consistently identified core insect-specific viruses alongside a vast array of plant-, fungi-, and protist-associated viral sequences [58,59].

The presence of these non-arthropod viruses is a common ecological signature of phlebotomines, directly reflecting their terrestrial breeding sites and diverse feeding habits. This is clearly illustrated by the detection of viruses belonging to the families *Partitiviridae*, *Narnaviridae*, *Tymoviridae*, and *Solemoviridae* in *Pi. fischeri*, which likely stems from the constant contact of sand fly larvae with soil-dwelling fungi and plants in their organic-rich breeding sites [60,61,62]. Consistent with this, similar associations with fungal-associated *Partitiviridae* and *Narnaviridae* have been reported in wild-caught *Phlebotomus* and *Lutzomyia* populations, suggesting that these viral families represent stable, widespread components of the sand fly ecological virome rather than isolated occurrences.

A striking finding was the high abundance of unclassified viruses belonging to the unclassified RNA viruses ShiM 2016 group [45], originally described in a global arthropod virosphere survey. Although frequently detected in metagenomic studies, this group is not formally recognized by the ICTV, and its taxonomic position remains unresolved. Their high representation in *Pi. fischeri* suggests that these viruses may represent recurrent components of the viral community associated with this species, although their biological relevance remains unknown.

The identification of four highly divergent viral sequences belonging to the families *Partitiviridae* and *Narnaviridae* expands the currently known diversity of RNA viruses associated with Neotropical sand flies. The relatively low amino acid identities observed in comparisons with publicly available sequences, together with their phylogenetic placement and conserved RdRp motifs, support the interpretation that these viruses represent divergent lineages within their respective families. According to current ICTV demarcation criteria for *Partitiviridae* and *Narnaviridae*, the levels of sequence divergence observed are consistent with the possibility that these viruses represent previously undescribed taxa, although formal taxonomic classification will require additional genomic and biological data.

In addition to phylogenetic characterization, read remapping and abundance analyses revealed marked differences in the representation of viral genomes within the metatranscriptomic library. Among the identified viruses, PfPartitiV-2-SP showed the highest abundance, genome coverage, and number of mapped reads, whereas PfNarnaV-1-SP was represented by substantially fewer reads and lower coverage values. These differences may reflect distinct replication dynamics, persistence patterns, host associations, or differences in viral load within the analyzed sample. The genomic coverage obtained for the partitiviruses, coupled with the identification of fully intact open reading frames (ORFs) containing conserved RNA-dependent RNA polymerase (RdRp) motifs, supports the reliability of the assemblies and reduces the likelihood that these sequences represent assembly artifacts. The combination of high, uniform read coverage and structural genome integrity serves as a robust bioinformatic proxy for the authenticity of these viral infections.

The detection of PfNarnaV-1-SP despite nuclease treatment is particularly intriguing. Members of the family *Narnaviridae* are characterized as capsidless RNA viruses that lack a protective protein shell. Because our experimental design included enzymatic enrichment using TURBO DNase and an RNase cocktail to remove exogenous nucleic acids, exposed viral genomes would theoretically be susceptible to degradation. Nevertheless, a near-complete genome of PfNarnaV-1-SP was recovered and characterized through independent annotation, conserved motif analyses, and phylogenetic inference. Several non-exclusive hypotheses may explain this observation. First, narnavirus RNAs may occur in association with RNA-dependent RNA polymerase molecules, forming ribonucleoprotein complexes that provide partial protection against nuclease [63,64]. Second, viral RNA may be compartmentalized within intracellular structures, such as mitochondria, vesicles, or membrane-associated complexes, thereby limiting enzymatic accessibility prior to cell lysis [65]. Alternatively, a relatively high intracellular viral load may have contributed to the persistence of viral RNA throughout sample processing. Although these hypotheses remain speculative, they provide biologically plausible explanations for the recovery of a capsidless virus following nuclease treatment.

The characterization of the *Pi. fischeri* virome carries important implications for understanding *Leishmania* transmission, as this sand fly species is a recognized vector of the parasite in Neotropical regions. The potential influence of ISVs on sand fly vector competence for *Leishmania* has been increasingly discussed; however, no functional evidence was generated in the present study to support such interactions [66,67]. Recent studies have further reinforced the importance of metatranscriptomic approaches for understanding viral ecology in phlebotomine sand flies. In *Phlebotomus chinensis*, metatranscriptomic analyses revealed a complex community of viruses, bacteria, and other microorganisms, highlighting the diversity of biological interactions occurring within these insects [58]. In parallel, functional studies demonstrated that sand fly-derived cells activate RNA interference (RNAi)-mediated antiviral responses against *Toscana virus* infection, suggesting that ISVs may influence vector immune pathways [68]. More recently, single-cell transcriptomic analyses showed that *Leishmania* development within sand flies is highly heterogeneous, emphasizing the complexity of interactions among parasites, hosts, and associated microbiota [69].

Together, these findings indicate that virome characterization should be interpreted within a broader ecological framework, in which viral diversity, host immune responses, and parasite dynamics may collectively influence vector competence and transmission processes. Therefore, while any direct association between the viruses identified here and *Leishmania* transmission remains hypothetical and must be interpreted cautiously, our genomic survey identifies candidates and provides a critical baseline for future functional studies. Controlled infection assays, tissue tropism analyses, and longitudinal surveys will be necessary to determine whether ISVs influence parasite development, immune responses, or ecological fitness in *Pi. fischeri*.

This study has several limitations. First, the analysis was based on a single pool containing 21 individuals, which restricts the assessment of individual, spatial, and seasonal variation in the virome. Therefore, the virome described here should not be interpreted as representative of the broader *Pi. fischeri* population from the São Paulo region. Second, host-read depletion was not performed because no reference genome is currently available for this species, and therefore the proportion of viral reads should be interpreted with caution. Third, no experimental validation, such as RT-PCR or Sanger sequencing, was performed, and all viral genomes were identified through in silico assembly and annotation. Although multiple assembly strategies and independent validation tools were employed to reduce the likelihood of artifacts, experimental confirmation will be important in future studies. Finally, some low-frequency viral signals, such as the single read assigned to *Rotavirus A*, most likely represent environmental or laboratory contamination, a common occurrence in metatranscriptomic datasets.

Despite the implementation of stringent bioinformatic pipelines, metatranscriptomic approaches remain susceptible to environmental contamination and low-level cross-sample contamination. The recovery of four viral genomes with consistent genomic coverage substantially reduces the likelihood of assembly artifacts but does not eliminate this possibility entirely. Recent studies have emphasized that low-frequency contaminants may generate false-positive signals, particularly for highly divergent viruses and for viral groups that remain underrepresented in reference databases [45].

Furthermore, the absence of experimental validation, such as RT-PCR or Sanger sequencing, limits the level of confidence that can be assigned to genome completeness. In addition, this study was based on a single metatranscriptomic library generated from total RNA, which was designed for viral transcript discovery rather than small RNA profiling. Consequently, virus-derived small RNA analyses could not be performed using the available dataset. Although RT-PCR or RT-qPCR validation would provide additional experimental support for the assembled viral genomes, such experiments require additional biological material and were therefore beyond the scope of the present study.

Nevertheless, the combined evidence from de novo genome assembly, sequence similarity searches, conserved motif analyses, phylogenetic inference, read remapping, genome-wide coverage profiles, and transcript abundance estimates strongly supports the authenticity of the viral genomes reported here. Future studies including additional biological samples, small RNA sequencing, and experimental validation will be important to further investigate the prevalence, biological relevance, and replication of these viruses.

Taken together, this study expands current knowledge of viral diversity associated with Neotropical sand flies and highlights the utility of metatranscriptomic approaches for uncovering previously unrecognized viral lineages. By integrating genome assembly, taxonomic classification, phylogenetic inference, structural characterization, and abundance analyses, this work provides new insights into the diversity of RNA viruses associated with *Pi. fischeri*. These findings contribute to our understanding of virus–host associations in phlebotomine sand flies, expand the currently known diversity of RNA viruses associated with these insects, and provide a foundation for future ecological, evolutionary, and taxonomic investigations.

## 5. Conclusions

This study demonstrates that the virome of *Pintomyia fischeri* is highly diverse, characterized by a predominance of highly divergent and formally unclassified RNA virus lineages. Even within a geographically restricted setting, such as an urban zoological park, the characterization of these novel viral genomes underscores the complexity of ecological interactions among the vector, its hosts, and its viral microbiome. These findings reinforce the role of phlebotomine sand flies as key reservoirs of unrecognized viral diversity at neotropical interfaces between urban and wild environments.

Beyond significantly expanding the catalog of sand fly-associated viruses, this work highlights the power of metatranscriptomic approaches to uncover previously hidden viral lineages, offering new perspectives on the ecology and evolution of vector viromes. Although the biological relevance of these viruses warrants further experimental validation, our results raise critical hypotheses regarding the potential role of insect-specific or symbiotic viruses in modulating vector competence for *Leishmania* and influencing broader aspects of sand fly biology.

Taken together, these findings establish a robust foundation for future ecological, evolutionary, and taxonomic investigations into virus–host associations, contributing to a more comprehensive understanding of viral diversity in medically important vectors. In the long term, this knowledge provides strategic baseline data to support virological and epidemiological surveillance while enhancing our understanding of the ecological drivers involved in the emergence and maintenance of vector-borne pathogens.

## Figures and Tables

**Table 1:** Read mapping statistics and normalized abundance of the assembled viral genomes recovered from the Meta29 metatranscriptomic library of *Pintomyia fischeri*.

Virus	Genome Length (bp)	Genome Length (kb)	Assigned Reads	RPK	TPM	Percentage (%)
PfNarnaV-1-SP	2369	2.369	31	13.08	1589.92	0.16
PfPartitiV-1a-SP	1703	1.703	2358	1384.67	168,259.58	16.80
PfPartitiV-1b-SP	1672	1.672	1391	832.36	101,097.87	10.12
PfPartitiV-2-SP	1646	1.646	9875	5999.39	729,052.63	72.92
Total	—	—	13,655	8229.50	1,000,000.00	100.00

## Data Availability

The sequencing data generated from the Meta29 sample are publicly deposited in the NCBI Sequence Read Archive (SRA) under BioProject PRJNA1465927, BioSample SAMN59824430, and SRA SRR38603290. The sequences of the RdRp coding regions of the four viruses identified in this study are available in GenBank under the accession numbers PfPartitiV-1a-SP (PX992747), PfPartitiV-1b-SP (PX992748), PfPartitiV-2-SP (PX992749), and PfNarnaV-1-SP (PX992750).

## Supplementary Materials

The following supporting information can be downloaded at https://www.mdpi.com/article/10.3390/microorganisms14081723/s1: Table S1: Summary of the sampling sites from which *Pi. fischeri* individuals were collected for the metatranscriptomic analysis. The table includes geographic coordinates, capture environment (ground or canopy), number of individuals obtained at each site, and additional notes regarding trap height or specific conditions. A total of 21 sand flies were included in the final pooled sample; Table S2. Coverage and abundance statistics of the viral genomes assembled from the Meta29 metatranscriptomic library. The table summarizes genome length, mapped bases, mean sequencing coverage, coverage variability (standard deviation), and the number of reads assigned to coding sequences (CDSs) using featureCounts; Table S3: Global alignment statistics of the Meta29 metatranscriptomic library. This table summarizes the global alignment metrics obtained after mapping the filtered Meta29 reads to the assembled viral reference sequences, including read counts, mapping rates, coverage distribution, sequencing error profiles, and base composition; Table S4: Protein-level similarity statistics (BLASTp) of viral ORFs identified in the Meta29 library. This table summarizes amino acid–level similarity searches (BLASTp) for the predicted viral proteins encoded by the assembled contigs; Table S5: Conserved protein domains identified in viral ORFs using Pfam. This table summarizes the conserved domains detected in the predicted viral proteins using Pfam, including domain identifiers, descriptions, positions within the protein, and statistical significance (i-Evalue).

## References

[1] Forattini O.P. (1973). Entomologia Médica: Psychodidae, Phlebotominae, Leishmanioses, Bartonelose.

[2] Shimabukuro P.H., Da Silva T.R., Ribeiro F.O., Baton L.A., Galati E.A. (2010). Geographical distribution of American cutaneous leishmaniasis and its phlebotomine vectors (Diptera: Psychodidae) in the state of São Paulo, Brazil. Parasit. Vectors.

[3] Borges A.F., Gomes R.S., Ribeiro-Dias F. (2018). Leishmania (Viannia) guyanensis in tegumentary Leishmaniasis. Pathog. Dis..

[4] Da Silva Vieira T., Arango Duque G., Ory K., Gontijo C.M., Soares R.P., Descoteaux A. (2019). Leishmania braziliensis: Strain-Specific Modulation of Phagosome Maturation. Front. Cell. Infect. Microbiol..

[5] World Health Organization Leishmaniasis. https://www.who.int/news-room/fact-sheets/detail/leishmaniasis.

[6] Camargo-Neves V.L.F.D., Gomes A.D.C., Antunes J.L.F. (2002). Correlação da presença de espécies de flebotomíneos (Diptera: Psychodidae) com registros de casos da leishmaniose tegumentar americana no Estado de São Paulo, Brasil. Rev. Soc. Bras. Med. Trop..

[7] Galvis-Ovallos F., Da Silva M.D., Bispo G.B.D.S., De Oliveira A.G., Neto J.R.G., Malafronte R.D.S., Galati E.A.B. (2017). Canine visceral Leishmaniasis in the metropolitan area of São Paulo: *Pintomyia fischeri* as potential vector of *Leishmania infantum*. Parasite.

[8] Galvis-Ovallos F., Ueta A.E., Marques G.D.O., Sarmento A.M.C., Araujo G., Sandoval C., Tomokane T.Y., Da Matta V.L.R., Laurenti M.D., Galati E.A.B. (2021). Detection of *Pintomyia fischeri* (Diptera: Psychodidae) with *Leishmania infantum* (Trypanosomatida: Trypanosomatidae) Promastigotes in a Focus of Visceral Leishmaniasis in Brazil. J. Med. Entomol..

[9] Muniz L.H.G., Rossi R.M., Neitzke H.C., Monteiro W.M., Teodoro U. (2006). Estudo dos hábitos alimentares de flebotomíneos em área rural no sul do Brasil. Rev. Saúde Pública.

[10] Laroche L., Bañuls A.-L., Charrel R., Fontaine A., Ayhan N., Prudhomme J. (2024). Sand flies and Toscana virus: Intra-vector infection dynamics and impact on *Phlebotomus perniciosus* life-history traits. PLoS Negl. Trop. Dis..

[11] Da Rosa A.P.A.T., Tesh R.B., Pinheiro F.P., Da Rosa J.F.S.T., Peterson N.E. (1983). Characterization of Eight New Phlebotomus Fever Serogroup Arboviruses (Bunyaviridae: Phlebovirus) from the Amazon Region of Brazil. Am. J. Trop. Med. Hyg..

[12] Shaw J.J., de Rosa A.T., Cruz A.C., Vasconcelos P.F.d.C., Rangel E., Shaw J. (2018). Brazilian Phlebotomines as Hosts and Vectors of Viruses, Bacteria, Fungi, Protozoa (Excluding Those Belonging to the Genus Leishmania) and Nematodes. Brazilian Sand Flies.

[13] Nanfack Minkeu F., Vernick K.D. (2018). A Systematic Review of the Natural Virome of Anopheles Mosquitoes. Viruses.

[14] de Oliveira Guimarães L., Simoes R.F., Chagas C.R., de Menezes R.M., Silva F.S., Monteiro E.F., Holcman M.M., Bajay M.M., Pinter A., de Camargo-Neves V.L. (2021). Assessing Diversity, Plasmodium Infection and Blood Meal Sources in Mosquitoes (Diptera: Culicidae) from a Brazilian Zoological Park with Avian Malaria Transmission. Insects.

[15] Guimarães L.D., Ribeiro G.D., Da Couto R., Ramos E.D., Morais V.D., Telles-de-Deus J., Helfstein V.C., Santos J.M., Deng X., Delwart E. (2025). Exploring mosquito virome dynamics within São Paulo Zoo: Insights into mosquito-virus-environment interactions. Front. Cell. Infect. Microbiol..

[16] Forattini O.P. (2002). Culicidologia Médica.

[17] Consoli R.A.G.B., Lourenço-de-Oliveira R. (1994). Principais Mosquitos de Importância Sanitária no Brasil.

[18] Lane J. (1953). Neotropical Culicidae.

[19] Bolger A.M., Lohse M., Usadel B. (2014). Trimmomatic: A flexible trimmer for Illumina sequence data. Bioinformatics.

[20] Babraham Bioinformatics FastQC: A Quality Control Tool for High Throughput Sequence Data. https://www.bioinformatics.babraham.ac.uk/projects/fastqc/.

[21] Bushmanova E., Antipov D., Lapidus A., Prjibelski A.D. (2019). rnaSPAdes: A de novo transcriptome assembler and its application to RNA-Seq data. GigaScience.

[22] Li W., Godzik A. (2006). Cd-hit: A fast program for clustering and comparing large sets of protein or nucleotide sequences. Bioinformatics.

[23] Langmead B., Salzberg S.L. (2012). Fast gapped-read alignment with Bowtie 2. Nat. Methods.

[24] Danecek P., Bonfield J.K., Liddle J., Marshall J., Ohan V., Pollard M.O., Whitwham A., Keane T., McCarthy S.A., Davies R.M. (2021). Twelve years of SAMtools and BCFtools. GigaScience.

[25] Okonechnikov K., Conesa A., García-Alcalde F. (2016). Qualimap 2: Advanced multi-sample quality control for high-throughput sequencing data. Bioinformatics.

[26] Liao Y., Smyth G.K., Shi W. (2014). featureCounts: An efficient general purpose program for assigning sequence reads to genomic features. Bioinformatics.

[27] Seemann T. (2014). Prokka: Rapid prokaryotic genome annotation. Bioinformatics.

[28] Wang L., Wang S., Li W. (2012). RSeQC: Quality control of RNA-seq experiments. Bioinformatics.

[29] Buchfink B., Xie C., Huson D.H. (2015). Fast and sensitive protein alignment using DIAMOND. Nat. Methods.

[30] Huson D.H., Auch A.F., Qi J., Schuster S.C. (2007). MEGAN analysis of metagenomic data. Genome Res..

[31] National Center for Biotechnology Information BLAST: Basic Local Alignment Search Tool. https://blast.ncbi.nlm.nih.gov/Blast.cgi.

[32] Simmonds P., Adriaenssens E.M., Lefkowitz E.J., Oksanen H.M., Zerbini F.M., Alfenas-Zerbini P., Aylward F.O., Dempsey D.M., Freitas-Astúa J., Hendrickson R.C. (2026). Changes to virus taxonomy, the international code of virus classification and nomenclature, and the ICTV statutes ratified by the International Committee on Taxonomy of Viruses (2025). Arch. Virol..

[33] Katoh K., Standley D.M. (2013). MAFFT Multiple Sequence Alignment Software Version 7: Improvements in Performance and Usability. Mol. Biol. Evol..

[34] Rombel I.T., Sykes K.F., Rayner S., Johnston S.A. (2002). ORF-FINDER: A vector for high-throughput gene identification. Gene.

[35] National Center for Biotechnology Information NCBI Conserved Domain Search. https://www.ncbi.nlm.nih.gov/Structure/wrpsb-out/wrpsb.cgi.

[36] Kyoto Encyclopedia of Genes and Genomes MOTIF: Searching Protein Sequence Motifs. https://www.genome.jp/tools/motif/MOTIF.html.

[37] Marchal I. (2024). LucaProt reveals the diverse global RNA virome. Nat. Biotechnol..

[38] Babaian A., Edgar R. (2022). Ribovirus classification by a polymerase barcode sequence. PeerJ.

[39] SWISS-MODEL Interactive Workspace. https://swissmodel.expasy.org/interactive.

[40] Pettersen E.F., Goddard T.D., Huang C.C., Couch G.S., Greenblatt D.M., Meng E.C., Ferrin T.E. (2004). UCSF Chimera—A visualization system for exploratory research and analysis. J. Comput. Chem..

[41] Kalyaanamoorthy S., Minh B.Q., Wong T.K.F., Von Haeseler A., Jermiin L.S. (2017). ModelFinder: Fast model selection for accurate phylogenetic estimates. Nat. Methods.

[42] Hoang D.T., Chernomor O., Von Haeseler A., Minh B.Q., Vinh L.S. (2018). UFBoot2: Improving the Ultrafast Bootstrap Approximation. Mol. Biol. Evol..

[43] FigTree. https://tree.bio.ed.ac.uk/software/figtree/.

[44] Hernández-Pelegrín L., Rodríguez-Gómez A., Abelaira A.B., Reche M.C., Crava C., Lim F.S., Bielza P., Herrero S. (2024). Rich diversity of RNA viruses in the biological control agent, Orius laevigatus. J. Invertebr. Pathol..

[45] Shi M., Lin X.D., Tian J.H., Chen L.J., Chen X., Li C.X., Qin X.C., Li J., Cao J.P., Eden J.S. (2016). Redefining the invertebrate RNA virosphere. Nature.

[46] Zanotto P.M., Gibbs M.J., Gould E.A., Holmes E.C. (1996). A reevaluation of the higher taxonomy of viruses based on RNA polymerases. J. Virol..

[47] Koonin E.V., Gorbalenya A.E., Chumakov K.M. (1989). Tentative identification of RNA-dependent RNA polymerases of dsRNA viruses and their relationship to positive strand RNA viral polymerases. FEBS Lett..

[48] Kao C.C., Singh P., Ecker D.J. (2001). De Novo Initiation of Viral RNA-Dependent RNA Synthesis. Virology.

[49] Hong Y., Cole T.E., Brasier C.M., Buck K.W. (1998). Evolutionary relationships among putative RNA-dependent RNA polymerases encoded by a mitochondrial virus-like RNA in the Dutch elm disease fungus, Ophiostoma novo-ulmi, by other viruses and virus-like RNAs and by the Arabidopsis mitochondrial genome. Virology.

[50] Gorbalenya A.E., Pringle F.M., Zeddam J.-L., Luke B.T., Cameron C.E., Kalmakoff J., Hanzlik T.N., Gordon K.H.J., Ward V.K. (2002). The Palm Subdomain-based Active Site is Internally Permuted in Viral RNA-dependent RNA Polymerases of an Ancient Lineage. J. Mol. Biol..

[51] Te Velthuis A.J.W. (2014). Common and unique features of viral RNA-dependent polymerases. Cell. Mol. Life Sci..

[52] Selisko B., Papageorgiou N., Ferron F., Canard B. (2018). Structural and Functional Basis of the Fidelity of Nucleotide Selection by Flavivirus RNA-Dependent RNA Polymerases. Viruses.

[53] Xu X. (2003). Molecular model of SARS coronavirus polymerase: Implications for biochemical functions and drug design. Nucleic Acids Res..

[54] Ng K.K.-S., Arnold J.J., Cameron C.E., Paddison P.J., Vogt P.K. (2008). Structure-Function Relationships Among RNA-Dependent RNA Polymerases. RNA Interference.

[55] Vázquez A.L., Alonso J.M.M., Parra F. (2000). Mutation Analysis of the GDD Sequence Motif of a Calicivirus RNA-Dependent RNA Polymerase. J. Virol..

[56] Tong L., Duan Y., Zhang W., Jiang B., Zeng M., Wang M., Jia R., Zhu D., Liu M., Zhao X. (2021). Motif C in nonstructural protein 5 of duck Tembusu virus is essential for viral proliferation. Vet. Microbiol..

[57] Nguyen L.-T., Schmidt H.A., Von Haeseler A., Minh B.Q. (2015). IQ-TREE: A Fast and Effective Stochastic Algorithm for Estimating Maximum-Likelihood Phylogenies. Mol. Biol. Evol..

[58] Wang J., Gou Q.-Y., Luo G.-Y., Hou X., Liang G., Shi M. (2022). Total RNA sequencing of Phlebotomus chinensis sandflies in China revealed viral, bacterial, and eukaryotic microbes potentially pathogenic to humans. Emerg. Microbes Infect..

[59] McCarthy C.B., Diambra L.A., Rivera Pomar R.V. (2011). Metagenomic Analysis of Taxa Associated with Lutzomyia longipalpis, Vector of Visceral Leishmaniasis, Using an Unbiased High-Throughput Approach. PLoS Negl. Trop. Dis..

[60] Moncaz A., Faiman R., Kirstein O., Warburg A. (2012). Breeding Sites of *Phlebotomus sergenti*, the Sand Fly Vector of Cutaneous Leishmaniasis in the Judean Desert. PLoS Negl. Trop. Dis..

[61] Feliciangeli M.D. (2004). Natural breeding places of phlebotomine sandflies. Med. Vet. Entomol..

[62] Souza T.L., Figueiredo F.B., Almeida A.B., Benigno C.V., Pontes C.S., Souza M.B. (2014). Natural breeding sites of phlebotomine sand flies (Diptera: Psychodidae) on Marambaia Island, Rio de Janeiro State, Brazil. Acta Trop..

[63] Hillman B.I., Cai G. (2013). The Family Narnaviridae. Advances in Virus Research.

[64] Wolf Y.I., Kazlauskas D., Iranzo J., Lucía-Sanz A., Kuhn J.H., Krupovic M., Dolja V.V., Koonin E.V. (2018). Origins and Evolution of the Global RNA Virome. mBio.

[65] Conceição-Neto N., Zeller M., Lefrère H., De Bruyn P., Beller L., Deboutte W., Yinda C.K., Lavigne R., Maes P., Ranst M.V. (2015). Modular approach to customise sample preparation procedures for viral metagenomics: A reproducible protocol for virome analysis. Sci. Rep..

[66] Tan L., Zhang Y., Kim D.Y., Li R. (2023). Insect-Specific Chimeric Viruses Potentiated Antiviral Responses and Inhibited Pathogenic Alphavirus Growth in Mosquito Cells. Microbiol. Spectr..

[67] De Faria I.J.S., De Almeida J.P.P., Marques J.T. (2024). Impact of symbiotic insect-specific viruses on mosquito vector competence for arboviruses. Curr. Opin. Insect Sci..

[68] Alexander A.J., Salvemini M., Sreenu V.B., Hughes J., Telleria E.L., Ratinier M., Arnaud F., Volf P., Brennan B., Varjak M. (2023). Characterisation of the antiviral RNA interference response to Toscana virus in sand fly cells. PLoS Pathog..

[69] Catta-Preta C.M.C., Ghosh K., Sacks D.L., Ferreira T.R. (2024). Single-cell atlas of Leishmania development in sandflies reveals the heterogeneity of transmitted parasites and their role in infection. Proc. Natl. Acad. Sci. USA.

