# Supplementary material for: Highly Divergent Partiti-like and Narna-like Viral RNA Sequences Detected in Pintomyia fischeri from São Paulo, Brazil: An Exploratory Metatranscriptomic Report
Source: Microorganisms. 2026 Aug 5;14(8):1723. doi: 10.3390/microorganisms14081723 (PMC13515979; doi:10.3390/microorganisms14081723)
Supplement: Supplementary file 1 [file microorganisms-14-01723-s001.zip › microorganisms-4414717-supplementary.pdf]

Supplementary Tables

Supplementary Table S1

Summary of the sampling sites from which *Pi. fischeri* individuals were collected for the metatranscriptomic analysis. The table includes geographic coordinates, capture environment (ground or canopy), number of individuals obtained at each site, and additional notes regarding trap height or specific conditions. A total of 21 sand flies were included in the final pooled sample.

Sampling site (ID)	Coordinates	Environment (Ground/Canopy)	Number of individuals	Notes
Ponte do Lago	23°39'08.1"S 46°37'03.9"W	Canopy	1	6.5m
Corredor 61	23°39'01.5"S 46°37'03.0"W	Ground / Canopy	5	1.5 m / 10 m
Recinto 69	23°39'11.2"S 46°36'59.7"W	Canopy	1	8.0m
Extra	23°38'48.2"S 46°37'14.4"W	Ground	13	1.5 m
Recinto 113	23°38'58.3"S 46°37'03.9"W	Canopy	1	7.5m
**Total**	—	—	**21**	—

Supplementary Table S2

Supplementary Table S2. Coverage and abundance statistics of the viral genomes assembled from the Meta29 metatranscriptomic library. The table summarizes genome length, mapped bases, mean sequencing coverage, coverage variability (standard deviation), and the number of reads assigned to coding sequences (CDSs) using featureCounts.

**Abundance and coverage statistics of viruses identified in the Meta29 library**					
**Viruses**	**Length (bp)**	**Mapped bases**	**Mean coverage (×)**	**Standard deviation**	**Assigned reads (featureCounts)**
PfNarnaV-1-SP	2,369	21,210	8.95	7.21	31
PfPartitiV-1a-SP	1,703	1,251,522	734.89	417.43	2,358
PfPartitiV-1b-SP	1,672	756,567	452.49	244.04	1,391
PfPartitiV-2-SP	1,646	5,095,434	3,095.65	1,679.91	9,875

Assigned reads correspond to CDS-associated read counts obtained with featureCounts, whereas mapped bases and coverage statistics were calculated from read remapping against complete viral genomes. Consequently, these metrics should not be interpreted as directly comparable because they quantify different genomic features.

Supplementary Table S3

Global alignment statistics of the Meta29 metatranscriptomic library. This table summarizes the global alignment metrics obtained after mapping the filtered Meta29 reads to the assembled viral reference sequences, including read counts, mapping rates, coverage distribution, sequencing error profiles, and base composition.

Parameter	Result
Alignment software	Bowtie2 v2.5.5
Alignment preset	--very-sensitive
Total filtered reads used for mapping	529,596
Mapped reads	26,909 (5.08%)
Unmapped reads	502,687 (94.92%)
Mapped paired reads	0 (0%)
Secondary alignments	0
Mean read Length	269.8 bp
Minimum/maximum read length	35–301 bp
Estimated duplication rate	77.55%
Duplicated reads (estimated)	24,152
Mean global coverage	964.10×
Coverage standard deviation	1,434.37
Mean mapping quality	30.09
General error rate	2.99%
Mismatches	195,065
Insertions	3,801
Mapped reads with ≥1 insertion	8.17%
Deletions	1,196
Mapped reads with ≥1 deletion	3.78%
Homopolymer indels	30.96%
A bases (%)	2,009,052 (28.21%)
C bases (%)	1,567,337 (22.00%)
T bases (%)	2,011,547 (28.24%)
G bases (%)	1,535,004 (21.55%)
N bases (%)	0 (0%)
GC contente	43.55%
Total viral reference size	7,390 bp

Supplementary Table S4

Protein-level similarity statistics (BLASTp) of viral ORFs identified in the Meta29 library. This table summarizes amino acid–level similarity searches (BLASTp) for the predicted viral proteins encoded by the assembled contigs.

Samplename	Proteins	Organism	Size (Base pairs)	Best-hit GenbankID	Coverage	E-values	Identity
PfPartitiV-1a-SP	RdRp	Partitiviridae sp.	494	WWV86526	99%	3×10^{-179}	53.27%
PfPartitiV-1b-SP	RdRp	Partitiviridae sp.	494	WWV86526	99%	7×10^{-177}	52.83%
PfPartitiV-2-SP	RdRp	Hubei partiti-like virus 57	493	APG78229.1	99%	4×10^{-180}	53.25%
PfNarnaV-1-SP	RdRp	Orius laevigatus narnavirus 1	786	XGU09087.1	93%	0×10^{0}	44.59%
	VP1 protein	Medvezhye Chrysops Narna-like virus 2	712	WQM60674.1	45%	1×10^{-17}	25.85%

Supplementary Table S5

Conserved protein domains identified in viral ORFs using Pfam. This table summarizes the conserved domains detected in the predicted viral proteins using Pfam, including domain identifiers, descriptions, positions within the protein, and statistical significance (i-Evalue).

Samplename	Pfam ID	Description	Pfam	Position	i-Evalue
**PfPartitiV-1a-SP**	PF00680	Viral RNA-dependent RNA polymerase	RdRP_1	57-441	2.8×10^{-48}
	PF02123	Viral RNA-directed RNA-polymerase	RdRP_4	294-403	1.3×10^{-6}
**PfPartitiV-1b-SP**	PF00680	Viral RNA-dependent RNA polymerase	RdRP_1	57-441	4.4×10^{-47}
	PF02123	Viral RNA-directed RNA-polymerase	RdRP_4	302-408	3×10^{-6}
**PfPartitiV-2-SP**	PF00680	Viral RNA-dependent RNA polymerase	RdRP_1	57-419	2.6×10^{-49}
	PF02123	Viral RNA-directed RNA-polymerase	RdRP_4	292-395	7.1×10^{-5}
**PfNarnaV-1-SP**	PF05919	Mitovirus RNA-dependent RNA polymerase	Mitovir_RNA_pol	372-519	4.2×10^{-07}
	PF23396	Family of unknown function (DUF7103) - VP1	DUF7103	439-493	1.1×10^{-1}
